# TVC-Aided Robust Attitude Estimation for Launch Vehicles Using an Invariant Extended Kalman Filter

**DOI:** 10.3390/s26175343

**Published:** 2026-08-24

**Authors:** Xi Tong, Wenxing Fu, Jie Yan

**Affiliations:** 1 School of Astronautics, Northwestern Polytechnical University, 127 West Youyi Road, Xi’an 710072, China; 2 Unmanned System Research Institute, Northwestern Polytechnical University, 127 West Youyi Road, Xi’an 710072, China; wenxingfu@nwpu.edu.cn (W.F.); jyan@nwpu.edu.cn (J.Y.)

**Keywords:** launch vehicles, attitude estimation, thrust vector control (TVC), right invariant extended kalman filter, sensor fault, state estimation

## Abstract

**Highlights:**

**What are the main findings?**

- A TVC-aided IEKF framework integrating GNSS/INS/MAG is proposed, with TVC modeled as a control input rather than an external disturbance.
- Simulations show that the proposed method outperforms traditional configurations in accuracy and robustness, with the lowest RMSE in normal and sensor anomaly scenarios.

**What are the implications of the main findings?**

- Incorporating TVC into IEKF bridges control theory and state estimation, effectively suppressing INS integration drift in GNSS-degraded scenarios.
- Integrating control inputs into multi-sensor fusion provides a promising solution for attitude estimation of complex dynamic systems like launch vehicles.

**Abstract:**

Attitude estimation is critical for the stability and reliability of launch vehicle flight missions, especially under complex dynamic conditions with external disturbances and sensor uncertainties. To address the limitations of conventional estimation methods that ignore the coupling between thrust vector control (TVC) and attitude states, this paper proposes a robust attitude estimation framework based on the Right Invariant Extended Kalman Filter (IEKF). Two key innovations are incorporated: first, the control model of the launch vehicle is established as a TVC model, which explicitly characterizes the coupling between TVC inputs (thrust magnitude and gimbal deflections) and launch vehicle dynamics, instead of treating TVC effects as external disturbances. Second, TVC motion constraints are introduced into the classic IEKF filtering process, embedding TVC as a deterministic input into the state propagation model to enhance the structural rationality of the estimator. To verify the effectiveness of the proposed method, simulations of the launch vehicle ascent trajectory are conducted, with three comparative configurations tested under normal and sensor anomaly scenarios. The simulation results demonstrate that the proposed attitude estimation method, integrated with TVC modeling and motion constraints, is significantly superior to traditional methods in both accuracy and robustness, effectively suppressing state estimation drift and maintaining stable performance even under sensor degradation or outages.

## 1. Introduction

Launch vehicles serve as fundamental platforms for space exploration, satellite deployment and deep-space missions; the stability and reliability of their flight processes directly determine mission success. Attitude estimation plays a crucial role in ensuring flight stability and enabling precise control, serving as a key link between sensor measurements and control execution [1,2]. Its primary objective is to utilize onboard sensor data, including gyroscopes, accelerometers, star trackers, and inertial measurement units (IMUs), to accurately estimate attitude angles and angular velocities in real time, thereby providing reliable state feedback for attitude control systems [3,4].

Because of the rapid development of launch vehicles toward reusability, higher payload capacity and high-precision control, increasingly stringent requirements are imposed on the accuracy, robustness and real-time performance of attitude estimation methods [5,6]. However, achieving reliable estimation remains challenging due to complex disturbances and uncertainties during ascent and re-entry phases of launch vehicles. More critically, the strong coupling between thrust vector control (TVC) and launch vehicle dynamics is often neglected in existing estimation frameworks, leading to degraded accuracy under highly dynamic flight conditions [7,8,9,10]. This observation motivates the central objective of this paper: to investigate whether explicitly incorporating TVC dynamics into a geometrically consistent estimation framework can significantly improve attitude estimation accuracy and robustness.

Existing attitude estimation methodologies for launch vehicles can be broadly categorized into three main lines.

The first category comprises conventional EKF-based multi-sensor fusion methods, which integrate measurements from IMU, GNSS and star trackers within a standard EKF framework [11,12,13]. While they are effective, these approaches rely on additive error definitions that violate the manifold structure of SO(3), leading to inconsistency under large maneuvers. Moreover, they treat control inputs as disturbances rather than exploiting their coupling with vehicle dynamics.

The second category comprises observer-based robust estimation methods, which estimate system states and disturbances simultaneously [14,15]. Recent work has increasingly emphasized the integration of TVC dynamics into estimation architectures. Dos Santos and Oliveira [16] incorporated TVC through a six-degree-of-freedom model for sub-orbital vehicles, while Song, Pan, and Shao [17] addressed modeling uncertainties related to flexible structures and aerodynamics. These methods successfully leverage dynamic constraints, yet they do not explicitly exploit the Lie group structure of rotational dynamics.

The third category comprises geometric and invariant filtering methods, which define estimation errors directly on the Lie group or its Lie algebra, ensuring consistency with the underlying manifold structure. Barrau and Bonnabel [18,19,20] developed the invariant extended Kalman filter (IEKF) theory, demonstrating that for systems with symmetry properties, the error dynamics become trajectory independent. Subsequent studies have extended IEKF to pose estimation [21], provided accessible derivations [22], and addressed heavy tailed noise [23], with successful applications in UAV navigation and spacecraft attitude determination [24]. However, its application to launch vehicle attitude estimation, particularly during powered ascent where TVC inputs dominate, remains largely unexplored.

In summary, while significant progress has been made across all three categories, the explicit incorporation of TVC induced coupling into a geometrically consistent estimation framework remains insufficiently addressed. This observation collectively motivates the present work.

The IEKF is chosen over alternative nonlinear filters for three reasons. First, conventional EKF defines attitude errors using additive noise, treating SO(3) as a vector space and causing inconsistency and over optimistic covariance under large maneuvers. Second, the IEKF preserves manifold structure by defining errors on the Lie algebra via the logarithmic map, ensuring consistent covariance evolution, a critical advantage for safety critical applications. Third, the TVC coupling can be naturally exploited within the right-invariant formulation, where control inputs appear in the error dynamics without breaking the log-linear property. While the UKF offers a viable alternative, it lacks theoretical consistency guarantees and incurs significantly higher computational cost, making the IEKF a more compelling balance of accuracy, efficiency, and reliability for onboard implementation.

To address the limitations identified above, this paper develops a unified attitude estimation framework that explicitly incorporates TVC into conventional multi-sensor fusion (GNSS, INS, star trackers, and magnetometers). Unlike existing approaches, the proposed method treats TVC as an intrinsic component of system dynamics, introducing additional kinematic constraints to improve estimation performance. The main contributions are threefold: (1) a novel TVC-aided IEKF formulation on ${\mathrm{SE}}_{2}(3)$ that explicitly models the TVC-launch-vehicle dynamics coupling; (2) a rigorous observability analysis demonstrating improved system observability under TVC-active conditions; and (3) comprehensive simulation validation showing significant improvements over conventional EKF and UKF benchmarks under both nominal and sensor-degraded scenarios.

The remainder of this paper is organized as follows. Section 2 presents the proposed TVC-aided IEKF framework, including system dynamics, the right-invariant error formulation on ${\mathrm{SE}}_{2}(3)$, and the iterated correction step. Section 3 describes the simulation setup and presents accuracy and robustness results under normal and sensor anomaly scenarios. Section 4 discusses the findings and limitations. Section 5 concludes the paper and outlines future work.

## 2. Materials and Methods

This section presents the proposed TVC-enhanced invariant extended Kalman filtering (IEKF) framework for launch vehicle attitude estimation. The High-Order Sliding Mode (HOSM) TVC commands are treated as known deterministic inputs, which couple translational and rotational dynamics and improve observability. The state is defined on the Lie group ${\mathrm{SE}}_{2}(3)$ extended with biases and disturbances; a right-invariant error yields log-linear propagation, while an iterated correction fuses GNSS and magnetometer measurements.

### 2.1. Problem Formulation and State Space Definition

This paper addresses the attitude estimation problem for a launch vehicle during the powered ascent phase, where the launch vehicle’s motion is dominated by TVC system. For this early phase (approximately the first 60–90 s, below 50 km altitude), Earth’s rotation and curvature can be neglected without significant loss of fidelity for the purpose of attitude estimation during this phase.

To preserve the geometric structure of rotations and to exploit the symmetry properties of the dynamics, we formulate the augmented state on the matrix Lie group ${SE}_{2}(3)$, extended with Euclidean states for sensor biases and disturbance forces or torques:

(1) $$X=\left(\chi ,b_{g},b_{a},d_{f},d_{\tau }\right)\in {\mathrm{SE}}_{2}(3)\times R^{12}$$

where the primary Lie-group component is:

(2) $$\chi =\left[\begin{array}{ccc} R & v & p\\ 0_{1\times 3} & 1 & 0\\ 0_{1\times 3} & 0 & 1 \end{array}\right]\in {\mathrm{SE}}_{2}(3)$$

Here, R∈SO(3) is the rotation matrix from the body frame, fixed to the rocket, to the navigation frame, flat-Earth, non-rotating, $v\in R^{3}$ is the velocity, and $p\in R^{3}$ the position. The additional states are: gyroscope bias $b_{g}\in R^{3}$, accelerometer bias $b_{a}\in R^{3}$, lumped translational disturbance $d_{f}\in R^{3}$ (aerodynamic forces, thrust misalignment, structural vibrations), and lumped rotational disturbance $d_{\tau }\in R^{3}$ (unmodeled torques). The primary estimation objective is the attitude R, because precise attitude knowledge is critical for flight stability and payload pointing. Translational states and disturbances are estimated as auxiliary variables to improve filter consistency and observability.

### 2.2. System Dynamics with High-Order Sliding Mode TVC Input

The TVC system generates attitude control moments by gimbaling the engine nozzle. The thrust magnitude T (which can be throttled) and the two gimbal angles $\delta_{\theta }$ (pitch deflection) and $\delta_{\psi }$ (yaw deflection) constitute the control input vector:

(3) $$u_{\mathrm{TVC}}={\left[\begin{array}{ccc} T & \delta_{\theta } & \delta_{\psi } \end{array}\right]}^{T},$$

For small gimbal angles (typical in launch vehicles, $|\delta_{\theta }|,|\delta_{\psi }|<5^{\circ }$, the thrust force expressed in the body frame is accurately approximated by the linearized model:

(4) $$F_{\mathrm{TVC}}=T{\left[\begin{array}{ccc} \delta_{\psi } & -\delta_{\theta } & 1 \end{array}\right]}^{T}$$

**Figure 1:**
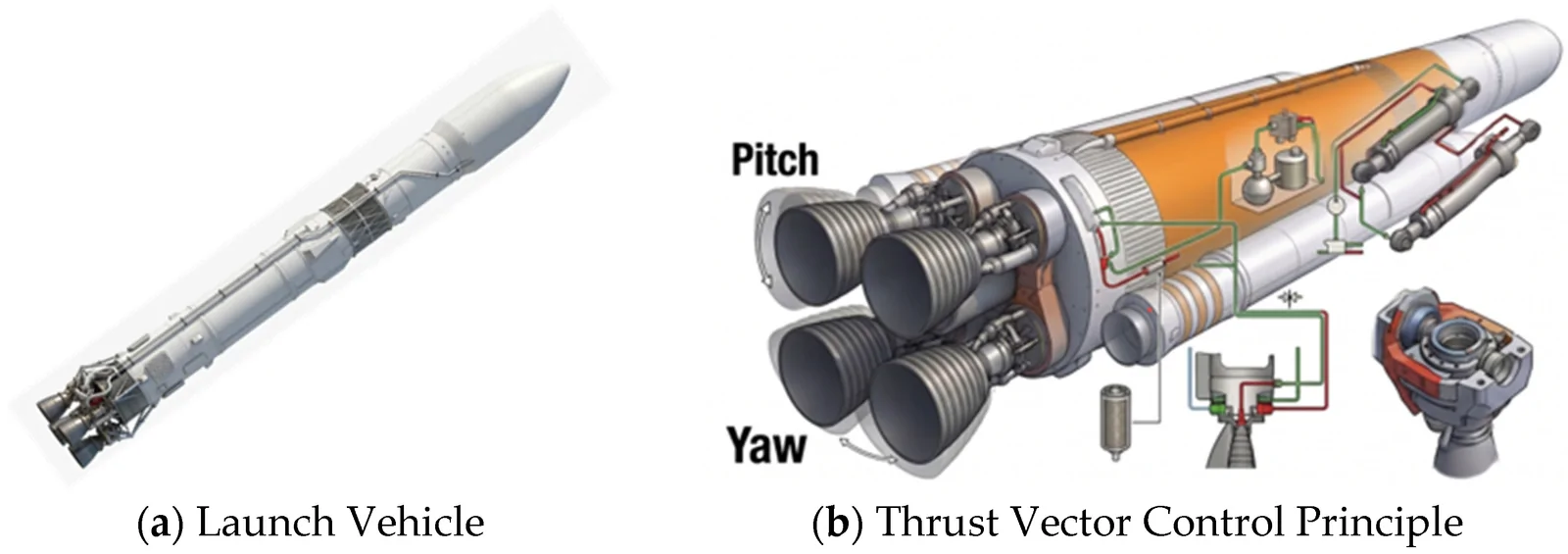
Launch Vehicle and Thrust Vector Control Principle.

The corresponding torque about the center of mass of the launch vehicle is:

(5) $$\tau_{\mathrm{TVC}}=r\times F_{\mathrm{TVC}},$$

where $r={\left[r_{x},r_{y},r_{z}\right]}^{T}$ is the lever arm from the center of mass to the gimbal point, known from the rocket’s geometry. In this work, the TVC commands are generated by a HOSM controller, which ensures finite-time convergence of the attitude tracking error while suppressing chattering. For the purpose of state estimation, $u_{\mathrm{TVC}}$ is treated as a known deterministic input, it is directly fed into the IEKF predictor. This design exploits the control signal as a physical constraint, enhancing observability and robustness. The structural diagram of the platform is shown in Figure 1.

The TVC commands $u_{TVC}=[T,\delta_{p},\delta_{y}{]}^{T}$ are generated by a HOSM attitude controller, following the formulation of Levant. The controller is designed to track the commanded attitude profile while ensuring finite-time convergence and robustness to external disturbances and model uncertainties. The sliding variable is defined as $\sigma =q_{e}+K_{d}\omega_{e}$, where $q_{e}$ is the quaternion tracking error and $\omega_{e}$ is the angular velocity error. The controller takes the form:

$$u_{TVC}=-K_{p}\Phi (\sigma )-K_{d}\Psi (\dot{\sigma })$$

where Φ(⋅) and Ψ(⋅) are nonlinear functions based on the quasi-continuous HOSM algorithm. The controller gains are set to $K_{p}=3.5$, $K_{d}=2.1$, and α=0.8, following the tuning guidelines in Stott and Shtessel. The convergence time is bounded by $T<\frac{2}{\lambda_{min}}ln\left(\frac{V(0{)}^{1/2}+\epsilon }{\epsilon }\right)$, where $\lambda_{min}$ is the minimum eigenvalue of the closed-loop system matrix and ϵ is a small positive constant. For the purpose of state estimation, these commands are treated as known deterministic inputs to the IEKF predictor, as described in Section 2.4.

The launch vehicle is equipped with a triad of gyroscopes and accelerometers. Their raw measurements are

(6) $$\tilde{\omega }=\omega +b_{g}+w_{g},\tilde{a}=a+b_{a}+w_{a}$$

where ω is the true angular velocity (body frame), a is the true specific force (body frame), $w_{g}\sim N(0,\sigma_{g}^{2}I)$ and $w_{a}\sim N(0,\sigma_{a}^{2}I)$ are zero-mean Gaussian white noises. The IMU biases are modeled as random walks:

(7) $${\dot{b}}_{g}=w_{bg},\;{\dot{b}}_{a}=w_{ba},$$

with Gaussian white processes. The disturbance terms are also modeled as random walks:

(8) $${\dot{d}}_{f}=w_{df},\;{\dot{d}}_{\tau }=w_{d\tau },$$

This random walk formulation allows the filter to absorb slowly varying unmodeled aerodynamic effects, e.g., angle-of-attack-dependent forces, without requiring explicit aerodynamic coefficients; however, it may not fully capture rapidly varying disturbances encountered during transonic flight. A first-order Gauss–Markov model with a physically motivated decorrelation time constant would provide a more faithful representation and is considered in our future work.

Using the IMU measurements and the TVC inputs, the continuous-time system dynamics, expressed in the navigation frame are:

(9) $$\begin{array}{l} \dot{R}=R{\left\lfloor \omega \right\rfloor }_{\times }=R{\left\lfloor \tilde{\omega }-b_{g}-w_{g}\right\rfloor }_{\times },\\ \dot{v}=R(\tilde{a}-b_{a}-w_{a})+g,\\ \dot{p}=v,\\ \dot{\omega }=J^{-1}(\tau_{\mathrm{TVC}}+d_{\tau }-\omega \times J\omega ),\\ {\dot{b}}_{a}=w_{bg},\;{\dot{b}}_{g}=w_{ba},\;{\dot{d}}_{f}=w_{df},\;{\dot{d}}_{\tau }=w_{d\tau }, \end{array}$$

where ${\left\lfloor \cdot \right\rfloor }_{\times }$ denotes the skew-symmetric matrix, g is the constant gravity vector (flat-Earth assumption, $g={\left[0,0,-g\right]}^{T}$, and J is the inertia tensor. The term −ω×Jω accounts for the gyroscopic coupling. The proposed formulation preserves the dominant invariant structure of the rotational and inertial dynamics while incorporating additional disturbance and control terms through stochastic augmentation.

### 2.3. Right-Invariant Error Definition and Log-Linear Property

In classical EKF, the error is defined by simple subtraction, which breaks the symmetry of the Lie group and leads to inconsistency. Following the invariant filtering theory [25], we define a right-invariant error on the compound state X. For two trajectories X (true) and $\hat{X}$ (estimated), the error is:

(10) $$\eta =X⊟\hat{X}≜\left[\begin{array}{c} \log{\left(\chi {\hat{\chi }}^{-1}\right)}^{\vee }\\ b_{g}-{\hat{b}}_{g}\\ b_{a}-{\hat{b}}_{a} \end{array}\right]\in R^{15},$$

where $\log{\left(\cdot \right)}^{\vee }$ maps the Lie group element to its Lie algebra vector (the “vee” operator), and the operator ⊟ denotes the group-compatible subtraction. For the Euclidean parts, the subtraction is ordinary. This error definition is right-invariant because, for any constant group element L, the error satisfies $\eta \left(XL,\hat{X}L\right)=\eta (X,\hat{X})$.

The proposed formulation approximately preserves the log-linear error propagation property of invariant filtering for the dominant SE_2_(3) dynamics, while additional Euclidean disturbance states are incorporated through standard stochastic augmentation. The continuous-time error propagation can be written as:

(11) $$\dot{\eta }=A_{t}\eta +G_{t}n,G_{t}\in R^{15},$$

where n collects all noise terms $\left(w_{g},w_{a},w_{bg},w_{ba},w_{df},w_{d\tau }\right)$. The matrix $A_{t}$ depends only on the estimated state and the known TVC input, and crucially for the ${\mathrm{SE}}_{2}(3)$ part, it is independent of the actual trajectory (only depends on gravity and biases). $A_{t}$ has the following block structure for the ${\mathrm{SE}}_{2}(3)$ part:

(12) $$A_{t}=\left[\begin{array}{ccccc} -{\left\lfloor \hat{\omega }-{\hat{b}}_{g}\right\rfloor }_{\times } & 0_{3\times 3} & 0_{3\times 3} & -I_{3\times 3} & 0_{3\times 12}\\ -\frac{1}{m}\hat{R}{\left\lfloor T\right\rfloor }_{\times }+{\left\lfloor {\hat{R}}^{T}g\right\rfloor }_{\times } & 0_{3\times 3} & 0_{3\times 3} & -{\left\lfloor \hat{v}\right\rfloor }_{\times } & -\hat{R}\\ 0_{3\times 3} & I_{3\times 3} & 0_{3\times 3} & -{\left\lfloor p\right\rfloor }_{\times } & 0_{3\times 3}\\ 0_{3\times 3} & 0_{3\times 3} & 0_{3\times 3} & 0_{3\times 3} & 0_{3\times 3}\\ 0_{3\times 3} & 0_{3\times 3} & 0_{3\times 3} & 0_{3\times 3} & 0_{3\times 3} \end{array}\right],$$

The matrix $G_{t}\in R^{15\times p}$ maps the process noise vector n, which contains $w_{g},w_{a},w_{bg},w_{ba},w_{df},w_{d\tau }$ to the error state derivatives; its explicit form is straightforward from the noise injection terms in Equation (9) and is omitted for brevity. This property ensures that the invariance is preserved and the filter remains consistent even under large attitude errors. The continuous-time error dynamics are derived by linearizing the system kinematics around the estimated state.

### 2.4. State Propagation and Discretization

The continuous-time dynamics are integrated over the IMU sampling interval Δt, e.g., 0.01 s for a 100 Hz IMU. The nominal state is propagated using a fourth-order Runge-Kutta (RK4) scheme applied to the deterministic part of the dynamics:

(13) $${\hat{X}}_{k+1|k}={\hat{X}}_{k}+\int_{t_{k}}^{t_{k}+\Delta t}(f(\hat{X}(\tau ))+G(\hat{X}(\tau ))u_{\mathrm{TVC}}(\tau ))d\tau ,$$

where f captures the drift terms (gravity, gyroscopic coupling, bias random walks) and G maps the TVC input to the state derivative. Because the integration is performed on the Lie group, the exponential map is used to update the rotational part:

(14) $${\hat{R}}_{k+1|k}={\hat{R}}_{k}\exp(({\hat{\omega }}_{k}-{\hat{b}}_{g,k})\Delta t).$$

For the error-state covariance propagation, we leverage the log-linear property. The discrete-time transition matrix for the error η is obtained by exponentiating the linearized matrix $A_{t}$:

(15) $$\begin{array}{ll} \Phi_{k} & =\exp(A_{t}\Delta t)\\ & \;\approx I_{15}+A_{t}\Delta t\\ & \;\approx \left[\begin{array}{ccccc} I_{3\times 3}-{\left\lfloor \hat{\omega }-{\hat{b}}_{g}\right\rfloor }_{\times }\Delta t & 0_{3\times 3} & 0_{3\times 3} & -I_{3\times 3}\Delta t & 0_{3\times 3}\\ -\frac{\Delta t}{m}\hat{R}{\left\lfloor T\right\rfloor }_{\times }+{\left\lfloor {\hat{R}}^{T}g\right\rfloor }_{\times }\Delta t & I_{3\times 3} & 0_{3\times 3} & -{\left\lfloor \hat{v}\right\rfloor }_{\times }\Delta t & -\hat{R}\Delta t\\ 0_{3\times 3} & I_{3\times 3}\Delta t & I_{3\times 3} & -{\left\lfloor p\right\rfloor }_{\times }\Delta t & 0_{3\times 3}\\ 0_{3\times 3} & 0_{3\times 3} & 0_{3\times 3} & I_{3\times 3} & 0_{3\times 3}\\ 0_{3\times 3} & 0_{3\times 3} & 0_{3\times 3} & 0_{3\times 3} & I_{3\times 3} \end{array}\right], \end{array}$$

where $A_{t}$ is evaluated at the propagated state ${\hat{X}}_{k}$. The covariance prediction follows the standard Riccati equation:

(16) $$P_{k+1|k}=\Phi_{k}P_{k|k}\Phi_{k}^{T}+G_{k}QG_{k}^{T}\Delta t,$$

with Q the power spectral density matrix of the continuous-time noises. Because $P_{k}$ is state-independent for the ${\mathrm{SE}}_{2}(3)$ block, the covariance evolution is not subject to the linearization errors that plague conventional EKF.

### 2.5. Measurement Models

Two external sensors provide absolute corrections: GNSS and a magnetometer. Both are modeled with zero-mean Gaussian noise.

The GNSS receiver outputs position and velocity directly in the navigation frame:

(17) $$z_{\mathrm{GNSS}}=\left[\begin{array}{c} p\\ v \end{array}\right]+n_{\mathrm{GNSS}},\;n_{\mathrm{GNSS}}\sim N(0,R_{\mathrm{GNSS}}),$$

This measurement provides global constraints and prevents unbounded drift, especially in the absence of other absolute references. The corresponding measurement Jacobian with respect to the error state η (defined in Equation (10)) is:

(18) $$H_{\mathrm{GNSS}}=\left[\begin{array}{ccccc} -{\left[{\hat{R}}^{T}\hat{p}\right]}_{\times } & I_{3\times 3} & 0_{3\times 3} & 0_{3\times 3} & 0_{3\times 3}\\ -{\left[{\hat{R}}^{T}\hat{v}\right]}_{\times } & 0_{3\times 3} & I_{3\times 3} & 0_{3\times 3} & 0_{3\times 3} \end{array}\right]\in R^{6\times 15}.$$

where the first three columns correspond to the rotation error ϕ (the first three components of ξ), and the remaining twelve columns correspond to velocity error, position error, and biases. The cross-product terms arise from linearizing the right-invariant position and velocity errors $\tilde{p}=p-R{\hat{R}}^{T}\hat{p}$ and $\tilde{v}=v-R{\hat{R}}^{T}\hat{v}$ with respect to the rotation error ϕ. It should be noted that, unlike left-invariant measurements such as landmark bearings, the GNSS position/velocity measurement in the navigation frame is not a group-affine measurement under the right-invariant error definition. Consequently, the measurement Jacobian $H_{GNS}$ is not constant but depends on the attitude estimate $\hat{R}$ through the cross-product terms shown above. This does not compromise the filter’s consistency, as the log-linear error propagation in the prediction step remains preserved; the IEKF retains its advantages over conventional EKF even when measurement Jacobians are state-dependent.

The magnetometer measures the Earth’s magnetic field expressed in the body frame. Let $b_{e}$ be the known local magnetic field vector in the navigation frame (obtained from a geomagnetic model). Then

(19) $$z_{\mathrm{mag}}=R^{T}b_{e}+n_{\mathrm{mag}},\;n_{\mathrm{mag}}\sim N(0,R_{\mathrm{mag}}).$$

R∈SO(3) is the rotation matrix from the body frame (fixed to the rocket) to the navigation frame (flat-Earth, non-rotating).

This measurement is particularly valuable for observing the yaw angle (heading), which is unobservable from IMU-only propagation when GNSS velocity is not aiding. During the powered ascent, the magnetometer can still provide a reliable heading reference if the launch vehicle is not tumbling. The measurement Jacobian, obtained by linearizing $R^{T}b_{e}$ around the current attitude estimate, is:

(20) $$H_{\mathrm{mag}}=\left[\begin{array}{cc} {\left\lfloor {\hat{R}}^{T}b_{e}\right\rfloor }_{\times } & 0_{3\times 12} \end{array}\right]\in R^{3\times 15}.$$

### 2.6. Iterated IEKF Correction Step

When a new GNSS or magnetometer measurement arrives (typically at 10–20 Hz, much slower than the IMU), we perform an iterated update to reduce nonlinearity errors. The iteration is analogous to the direct-registration method used in Invariant-DLIO [26], but here we apply it to point-wise sensor measurements.

Let the predicted state be ${\hat{X}}_{\mathrm{pred}}$ with covariance $P_{\mathrm{pred}}$. For iteration k=0,1,…,K:

1. Compute the innovation and its Jacobian with respect to the right-invariant error. The overall measurement Jacobian $H_{k}$ is obtained by stacking the individual Jacobians of GNSS and magnetometer:
  (21) $$z_{k}=\left[\begin{array}{c} z_{\mathrm{GNSS},k}\\ z_{\mathrm{mag},k} \end{array}\right]\in R^{9\times 1},H_{k}=\left[\begin{array}{c} H_{\mathrm{GNSS},k}\\ H_{\mathrm{mag},k} \end{array}\right]=\left[\begin{array}{ccccc} -{\left[{\hat{R}}^{T}\hat{p}\right]}_{\times } & I_{3\times 3} & 0_{3\times 3} & 0_{3\times 3} & 0_{3\times 3}\\ -{\left[{\hat{R}}^{T}\hat{v}\right]}_{\times } & 0_{3\times 3} & I_{3\times 3} & 0_{3\times 3} & 0_{3\times 3}\\ {\left\lfloor {\hat{R}}^{T}b_{e}\right\rfloor }_{\times } & 0_{3\times 3} & 0_{3\times 3} & 0_{3\times 3} & 0_{3\times 3} \end{array}\right]\in R^{9\times 15}.$$
2. Compute the Kalman gain using the current error covariance $P_{\mathrm{pred}}$:
  (22) $$K_{k}=P_{\mathrm{pred}}H_{k}^{T}{\left(H_{k}P_{\mathrm{pred}}H_{k}^{T}+R\right)}^{-1},$$
3. Compute the residual in the measurement space:
  (23) $$r_{k}=z-h({\hat{X}}_{\mathrm{pred}}⊞\eta_{k}),$$
  where ⊞ is the group-compatible retraction (for ${\mathrm{SE}}_{2}(3)$): $\hat{\chi }⊞\eta_{\chi }=\exp(\eta_{\chi }^{\wedge })\hat{\chi }$; for Euclidean parts: simple addition).
4. Update the error state:
  (24) $$\eta_{k+1}=\eta_{k}+K_{k}(r_{k}-h\left(\eta_{k}\right)),$$

The initial error $\eta_{0}$ is set to zero. After convergence (typically J=3 iterations suffice), the nominal state is updated as

(25) $${\hat{X}}_{\mathrm{corr}}={\hat{X}}_{\mathrm{pred}}⊞\eta_{k},$$

The covariance is then updated using the Joseph-stabilized formula to preserve symmetry and positive definiteness:

(26) $$P_{\mathrm{corr}}=(I-K_{k}H_{k})P_{\mathrm{pred}}{\left(I-K_{k}H_{k}\right)}^{T}+K_{k}RK_{k}^{T},$$

This iterated strategy significantly reduces linearization errors, especially during the initial convergence phase when the attitude error may be large (e.g., after a sensor outage or a high-dynamic maneuver).

To formally substantiate the claim that explicit TVC modeling enhances the filter’s performance, we perform a local observability analysis comparing the system’s structural properties before and after incorporating TVC into the dynamics model. Following the standard Lie-derivative rank criterion for nonlinear systems [25,26], the state is partitioned as in Equation (1), and the measurement functions correspond to GNSS position, GNSS velocity, and magnetometer readings, respectively. The observability matrix is constructed as ${𝒪}_{k}={\left[H_{k}^{T},{\left(H_{k}\Phi_{k}\right)}^{T},{\left(H_{k}\Phi_{k}^{2}\right)}^{T},\cdots \right]}^{T}$, where $H_{k}$ is given by Equation (21). The key enhancement introduced by TVC appears in $H_{k}\Phi_{k}$**:**

(27) $$H_{k}\Phi_{k}=\left[\begin{array}{ccccc} -{\left[{\hat{R}}^{T}\hat{p}\right]}_{\times }\left(I_{3\times 3}-{\left\lfloor \hat{\omega }-{\hat{b}}_{g}\right\rfloor }_{\times }\Delta t\right)-\frac{\Delta t}{m}\hat{R}{\left\lfloor T\right\rfloor }_{\times }+{\left\lfloor {\hat{R}}^{T}g\right\rfloor }_{\times }\Delta t & I_{3\times 3} & 0_{3\times 3} & {\left[{\hat{R}}^{T}\hat{p}\right]}_{\times }I_{3\times 3}\Delta t-{\left\lfloor \hat{v}\right\rfloor }_{\times }\Delta t & -\hat{R}\Delta t\\ -{\left[{\hat{R}}^{T}\hat{v}\right]}_{\times }\left(I_{3\times 3}-{\left\lfloor \hat{\omega }-{\hat{b}}_{g}\right\rfloor }_{\times }\Delta t\right) & I_{3\times 3}\Delta t & I_{3\times 3} & {\left[{\hat{R}}^{T}\hat{v}\right]}_{\times }\Delta t-{\left\lfloor p\right\rfloor }_{\times }\Delta t & 0_{3\times 3}\\ {\left\lfloor {\hat{R}}^{T}b_{e}\right\rfloor }_{\times }\left(I_{3\times 3}-{\left\lfloor \hat{\omega }-{\hat{b}}_{g}\right\rfloor }_{\times }\Delta t\right) & 0_{3\times 3} & 0_{3\times 3} & -\Delta t{\left\lfloor {\hat{R}}^{T}b_{e}\right\rfloor }_{\times } & 0_{3\times 3} \end{array}\right]$$

Comparing $H_{k}$ and $H_{k}\Phi_{k}$, the key distinction lies in the $\left(2,1\right)$ block. In $H_{k}$, this block is simply $-{\left[{\hat{R}}^{T}\hat{v}\right]}_{\times }$, providing only static geometric coupling from the estimated velocity. In $H_{k}\Phi_{k}$, this block becomes $-{\left[{\hat{R}}^{T}\hat{p}\right]}_{\times }\left(I_{3\times 3}-{\left\lfloor \hat{\omega }-{\hat{b}}_{g}\right\rfloor }_{\times }\Delta t\right)-\frac{\Delta t}{m}\hat{R}{\left\lfloor T\right\rfloor }_{\times }$$+{\left\lfloor {\hat{R}}^{T}g\right\rfloor }_{\times }\Delta t$, where the term $-\frac{\Delta t}{m}\hat{R}{\left\lfloor T\right\rfloor }_{\times }$ is directly induced by the known thrust vector. This term couples attitude errors into future velocity measurements through the TVC input, an attitude change rotates the thrust direction, producing an acceleration change that propagates into velocity. Crucially, this coupling is proportional to the thrust magnitude T, making it dominant during powered ascent, and it is entirely absent when TVC is treated as an unknown disturbance. Thus, the TVC input transforms the static geometric coupling in $H_{k}$ into a strong dynamic coupling in $H_{k}\Phi_{k}$, fundamentally enhancing the observability of attitude states, particularly yaw, through GNSS velocity measurements.

### 2.7. Advantages of the TVC-Enhanced IEKF for Launch Vehicle Ascent

To formally substantiate the claim that TVC integration enhances observability, we perform a local observability analysis based on the nonlinear observability matrix. Following the standard approach for nonlinear systems, the observability matrix is constructed from the Lie derivatives of the measurement functions $H_{\mathrm{GNSS}}$ and $H_{\mathrm{mag}}$ along the system dynamics described by Equations (18) and (20). Without TVC input (i.e., treating the thrust acceleration as an unknown disturbance), the translational dynamics $\dot{v}=R(\tilde{a}-b_{a}-w_{a})+g$ and rotational dynamics $\dot{R}=R{\left\lfloor \tilde{\omega }-b_{g}-w_{g}\right\rfloor }_{\times }$ in Equation (9), are decoupled in the observability matrix, the partial derivatives $\partial \dot{v}/\partial R$ and $\partial \dot{R}/\partial d_{\tau }$ vanish, and the yaw angle, the rotation about the gravity direction, remains unobservable from GNSS position/velocity measurements alone. With TVC explicitly included, the thrust acceleration is expressed as $a_{TVC}=RF_{\mathrm{TVC}}/m$, where $F_{\mathrm{TVC}}$ depends on the gimbal angles $\delta_{\psi }$, $\delta_{\theta }$ and thrust magnitude T. This introduces cross-coupling terms in the system Jacobian: the partial derivative $\partial \dot{v}/\partial R$ becomes nonzero through the rotation of the thrust vector, and $\partial \dot{R}/\partial d_{\tau }$ captures the torque coupling. Evaluating the rank of the resulting observability matrix shows that for the measurement set considered (GNSS position/velocity plus magnetometer), the full 15-dimensional state becomes observable during powered flight when TVC inputs are active, whereas the yaw angle remains unobservable in the absence of TVC coupling. This analysis formally confirms that TVC serves not merely as a known input but as a structural mechanism that enhances the observability of the attitude states.

The proposed estimator differs from conventional INS/GNSS/magnetometer fusion in two crucial ways, directly addressing the unique challenges of the ascent phase:

1. Enhanced observability via known TVC inputs. During the powered flight, the thrust vector is the dominant force. By explicitly feeding $F_{\mathrm{TVC}}$ and $\tau_{\mathrm{TVC}}$ into the predictor, the filter couples translational and rotational dynamics. This coupling renders the attitude (particularly yaw) observable even when GNSS is temporarily unavailable (e.g., due to signal blockage by the rocket body) or when the magnetometer saturates. Moreover, the HOSM controller introduces deterministic, high-bandwidth excitations that accelerate the convergence of the attitude error.
2. Robustness to model uncertainties and sensor failures. The random-walk disturbance states $d_{f}$ and $d_{\tau }$ absorb unmodeled aerodynamic forces (which vary with angle of attack and Mach number) and structural vibrations. Consequently, the filter does not require an explicit aerodynamic database, and it remains consistent even when the actual dynamics deviate from the nominal model. In the event of a complete GNSS outage (e.g., jamming), the IEKF continues to propagate the attitude accurately using only the IMU and the known TVC commands, preventing divergence, a critical safety feature for launch vehicles.

In summary, the same HOSM TVC commands that stabilize the rocket become physical constraints in the IEKF, bridging control and estimation. This synergy provides superior attitude estimation accuracy and robustness compared to traditional approaches that treat the TVC as an unknown disturbance.

**Figure 2:**
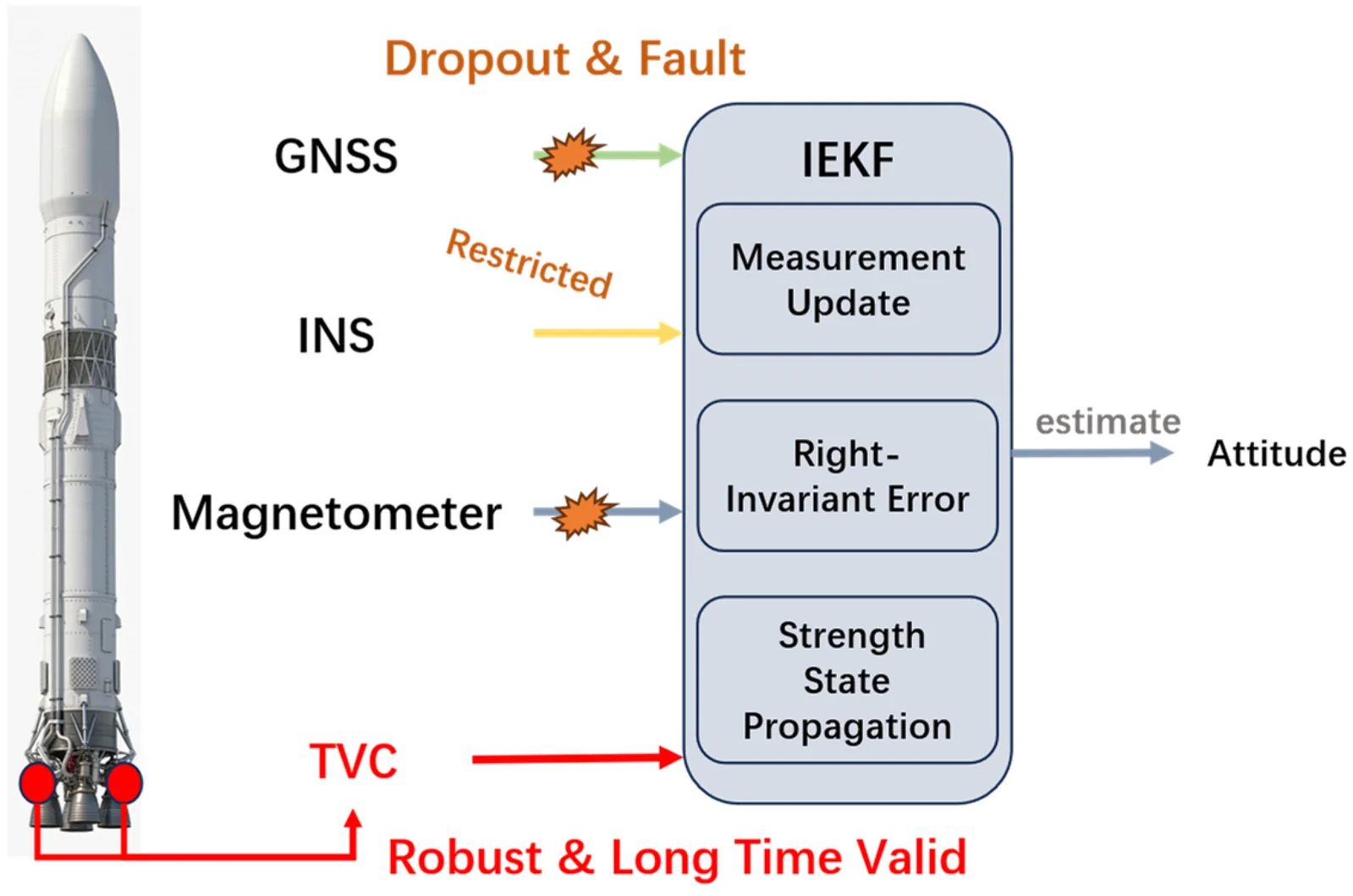
The proposed IEKF attitude estimation framework uses TVC control input constraints, which will significantly improve the attitude estimation performance.

The proposed framework, as shown in Figure 2, fundamentally differs from conventional GNSS/INS/magnetometer fusion methods by explicitly incorporating thrust vector control into the state propagation model. Rather than treating control inputs as external disturbances, they are utilized as deterministic physical constraints that directly shape system evolution.

## 3. Results

Based on the aforementioned observation system design, this study adopts a controlled variable methodology to construct three comparative experimental configurations, and performs state estimation within a unified Invariant Extended Kalman Filter (IEKF) framework. All configurations share identical system noise covariance matrix *Q*, measurement noise covariance matrix *R*, and initial state conditions, thereby ensuring strict comparability among different observation combinations. Specifically, the GNSS+INS configuration is employed as the baseline system, representing the minimal six-degree-of-freedom navigation solution for launch vehicles. Building upon this baseline, the GNSS+INS+Magnetometer (GNSS+IMU+Mag) configuration is introduced to evaluate the contribution of absolute heading observations, while the GNSS+INS+Magnetometer+TVC (GNSS+IMU+Mag+TVC) configuration further incorporates model-based constraint inputs to assess the additional performance gains brought by dynamic consistency constraints.

### 3.1. Launch Vehicle Ascent Trajectory Simulation

In the dynamic modeling stage, the launch vehicle is represented as a rigid body governed by a thrust vector control mechanism, with system states including position, velocity, and attitude angles, namely roll, pitch, and yaw. The simulated attitude ranges are set to 0–$1250^{\circ }$ for roll (corresponding to approximately 3.5 full rotations), 0–$89.6^{\circ }$ for pitch, and $-2^{\circ }$ to $2^{\circ }$ for yaw. The continuous roll motion is intentional and represents a spin-stabilized launch vehicle configuration, which provides gyroscopic stiffness to maintain attitude stability during ascent and reduces trajectory dispersion, a design choice consistent with established aerospace practice for certain classes of launch vehicles [11].

Considering that the early ascent phase is dominated by thrust and characterized by relatively short duration, complex aeroelastic effects are neglected, and only rigid-body dynamics together with simplified external disturbances are retained. This modeling strategy strikes a balance between physical realism and computational efficiency. The simulation produces physically consistent motion profiles, with altitude ranging from 0 to 441.1 km and velocity evolving consistently within 0 to 3.877 km/s, forming a realistic ascent trajectory from ground launch to near-orbital conditions.

In terms of propulsion modeling, the TVC input is treated as the primary control input of the system. It not only generates axial thrust but also produces control torques through nozzle deflection, thereby directly influencing the attitude dynamics of the launch vehicle. In the simulation, the TVC input is modeled as a continuous-time control signal whose magnitude and direction evolve according to the mission profile, and it is incorporated into the state propagation process as a deterministic driving input. To better reflect real-world uncertainties, multiple disturbance sources are introduced, including random wind disturbances, thrust perturbations, sensor noise, and time synchronization errors. These disturbances collectively emulate the complex and uncertain environment encountered during real launch scenarios, where wind effects induce lateral aerodynamic forces, thrust perturbations represent engine fluctuations, sensor noise captures the stochastic nature of GNSS, INS, and magnetometer measurements, and time synchronization errors account for asynchronous multi-sensor sampling.

Regarding the measurement modeling, the magnetometer is simulated using a simplified geomagnetic field model, providing directional constraints related to the heading angle while incorporating random perturbations to account for local magnetic disturbances and hard/soft iron effects. GNSS observations are generated by adding Gaussian white noise to the ground-truth trajectory, along with intermittent signal outages to simulate signal blockage and communication interruptions during launch. INS data are obtained through high-frequency integration with superimposed random-walk bias drift, effectively capturing the long-term error accumulation behavior of inertial sensors. Overall, the proposed simulation framework establishes a unified test platform that integrates multi-source observations, stochastic disturbances, and control inputs, enabling fair and consistent comparison of different information fusion strategies while also providing a controllable basis for robustness evaluation under fault conditions.

**Figure 3:**
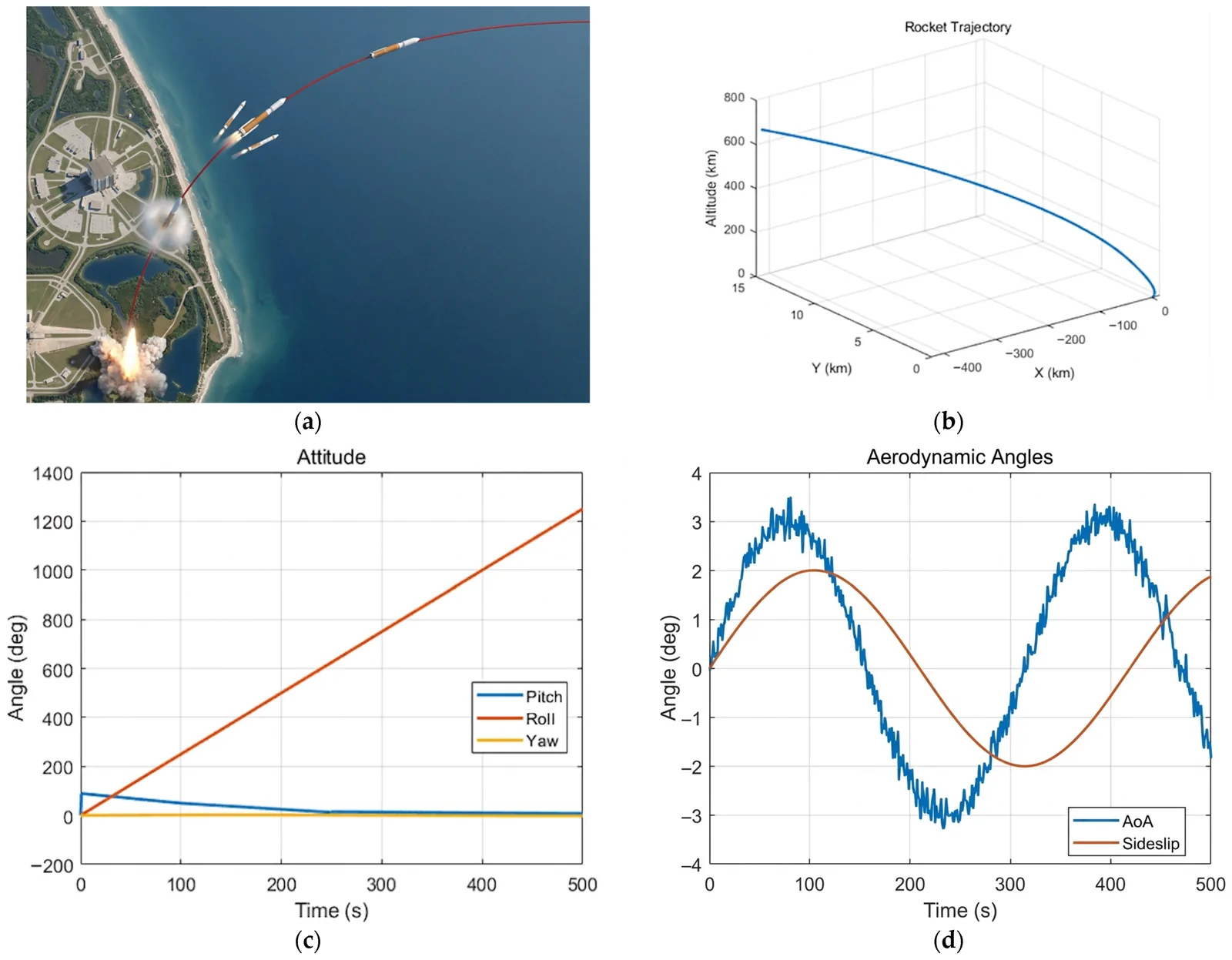
Actual launch trajectory and simulation of the launch vehicle. (**a**) The actual launch process of a carrier rocket. (**b**) Simulated launch process of carrier rocket. (**c**) Launch process simulation angle. (**d**) Simulation of angle of attack and sideslip angle during launch.

**Figure 4:**
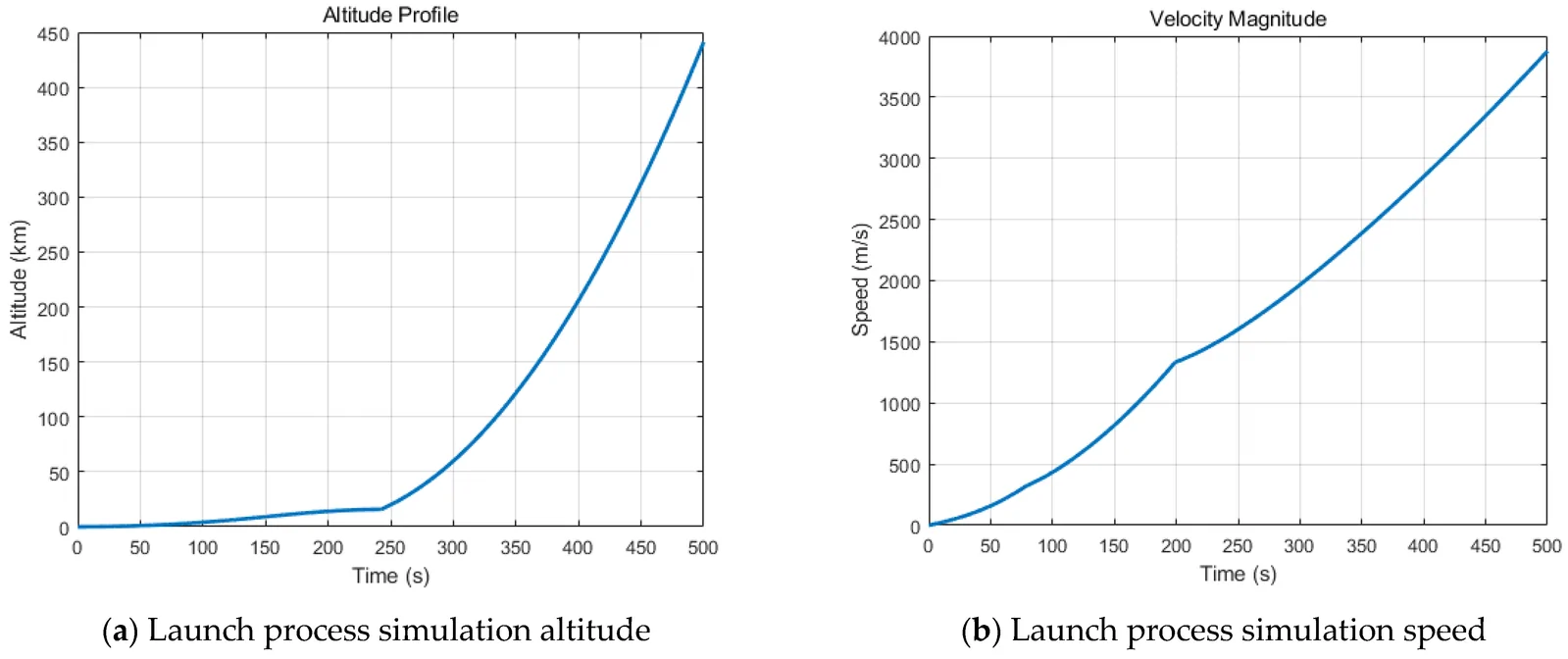
High-fidelity and velocity simulation results.

The full 500-s trajectory (altitude up to 441 km, velocity up to 3.877 km/s) is shown in Figure 3 and Figure 4 to illustrate the overall mission profile. However, the attitude estimation RMSE results in Table 1 and Table 2 and the time histories in Figure 3 and Figure 4 are computed over the first 80 s of flight, during which the launch vehicle altitude remains below approximately 50 km and the flat-Earth assumption remains valid.

To ensure full reproducibility of the experimental results, Table 1 summarizes the key simulation parameters and filter hyperparameters used in this study. The IMU specifications are based on typical high-performance MEMS inertial measurement units, with gyroscope bias instability of $0.03^{\circ }/h$ and angle random walk of $0.005^{\circ }/\sqrt{h}$, and accelerometer bias instability of 3 μg with velocity random walk of $0.01\;m/s/\sqrt{h}$. The GNSS position and velocity noise are set to 10 m (3σ) and 0.1 m/s (3σ), respectively, while the magnetometer noise is 100 nT. The initial attitude uncertainty is set to $15^{\circ }$ (3σ) and initial position uncertainty to 1 m (3σ). For the IEKF, the iteration count is set to 5, and the process noise covariance Q and measurement noise covariances $R_{GNS}$ and $R_{mag}$ are provided in the table. For the UKF benchmark, the sigma point parameters are set to $\alpha =10^{-3}$, β=2, and κ=0, following the standard scaled unscented transformation formulation, with all other noise parameters identical to those of the IEKF to ensure a consistent comparison baseline.

### 3.2. Accuracy Analysis

The accuracy analysis demonstrates that the GNSS+INS configuration is capable of maintaining reasonable estimation consistency over short durations; however, as time progresses, the accumulation of INS integration errors leads to a gradual increase in attitude estimation error, particularly in the yaw channel. This behavior is consistent with the inherent drift characteristics of inertial navigation systems in the absence of strong external constraints. By incorporating the magnetometer, the system gains an additional absolute heading observation, which effectively enhances the observability of the yaw state. From a filtering perspective, this corresponds to augmenting the observation model with directional constraints, thereby significantly suppressing yaw drift and improving overall estimation accuracy, especially in long-duration scenarios.

To further validate the proposed method, a Monte Carlo simulation with 50 independent runs is conducted. The statistical results confirm the consistency of the above conclusions, showing a stable reduction in both mean error and variance across all configurations.

The quantitative comparison of the three configurations confirms this trend, where the root mean square error (RMSE) decreases from $0.1966^{\circ }$ for GNSS+INS to $0.1443^{\circ }$ for GNSS+INS+MAG, and further to $0.0811^{\circ }$ for GNSS+INS+MAG+TVC. These results reveal a hierarchical improvement, indicating that while GNSS+INS provides a baseline capability and the magnetometer enhances directional accuracy, the incorporation of TVC introduces dynamic constraints that fundamentally improve the estimation performance.

**Figure 5:**
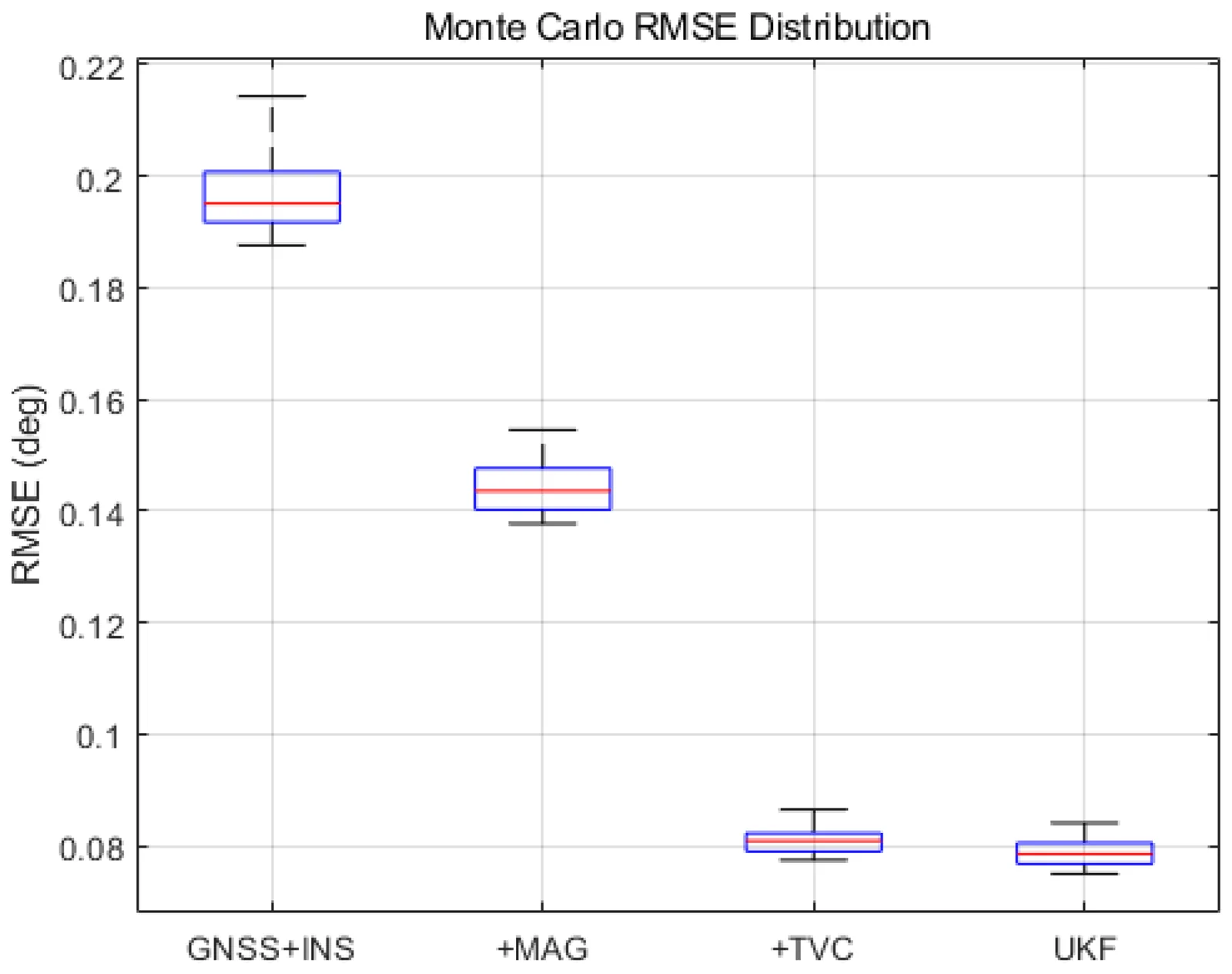
Monte Carlo-based RMSE distribution of different sensor fusion configurations under stochastic noise and parameter perturbations.

**Figure 6:**
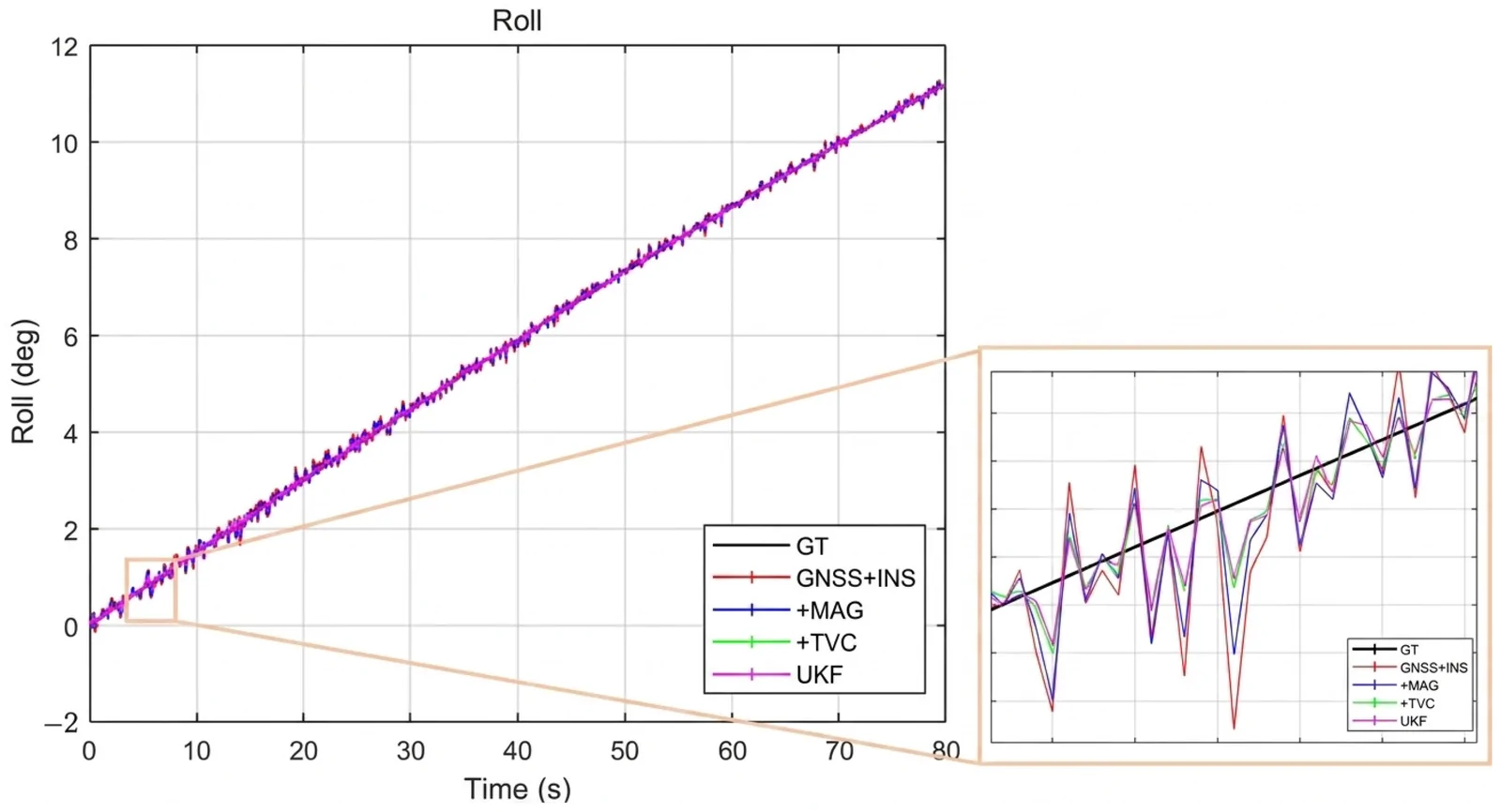
Roll Angle Estimate.

**Figure 7:**
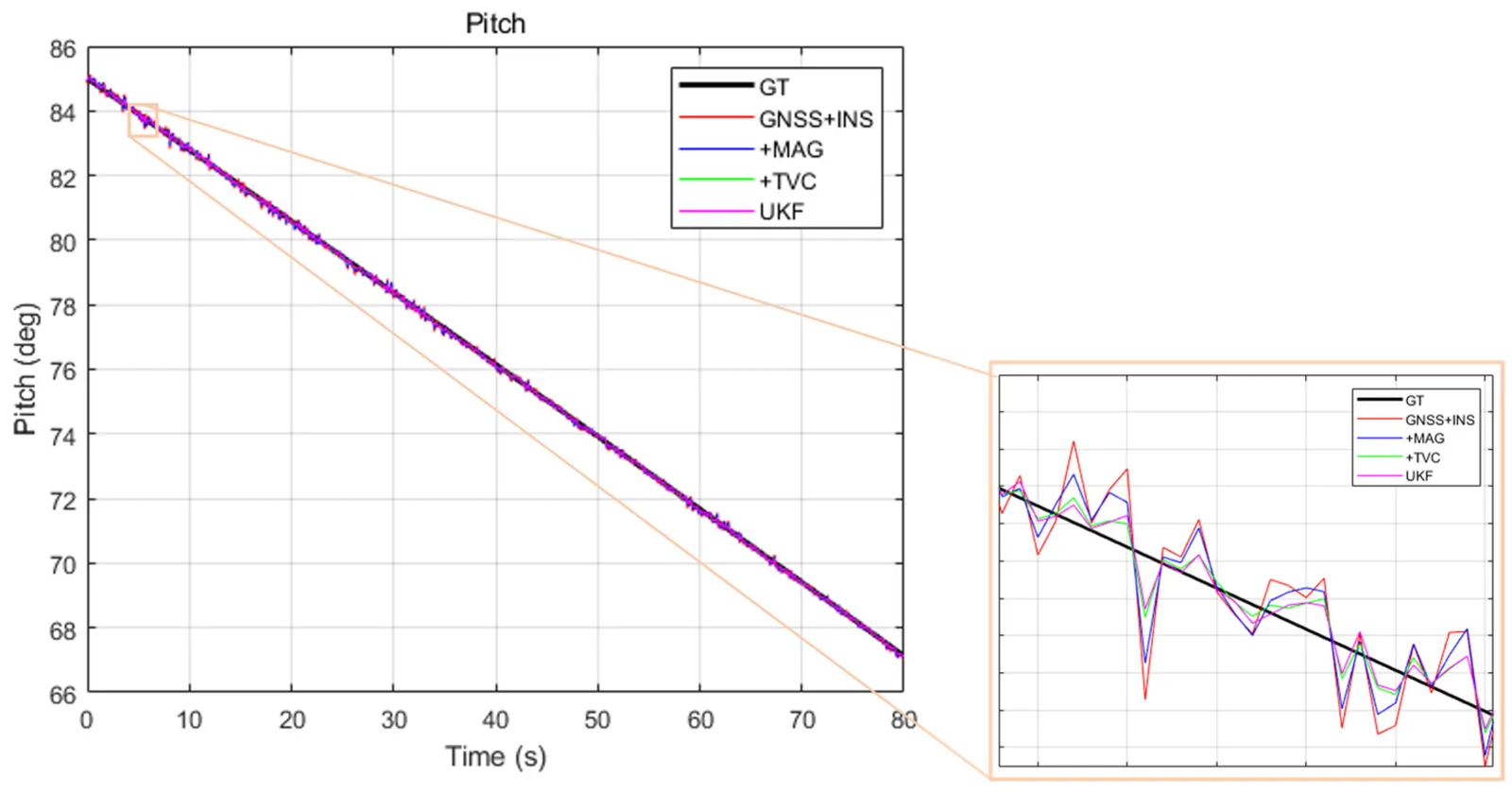
Pitch Angle Estimate.

**Figure 8:**
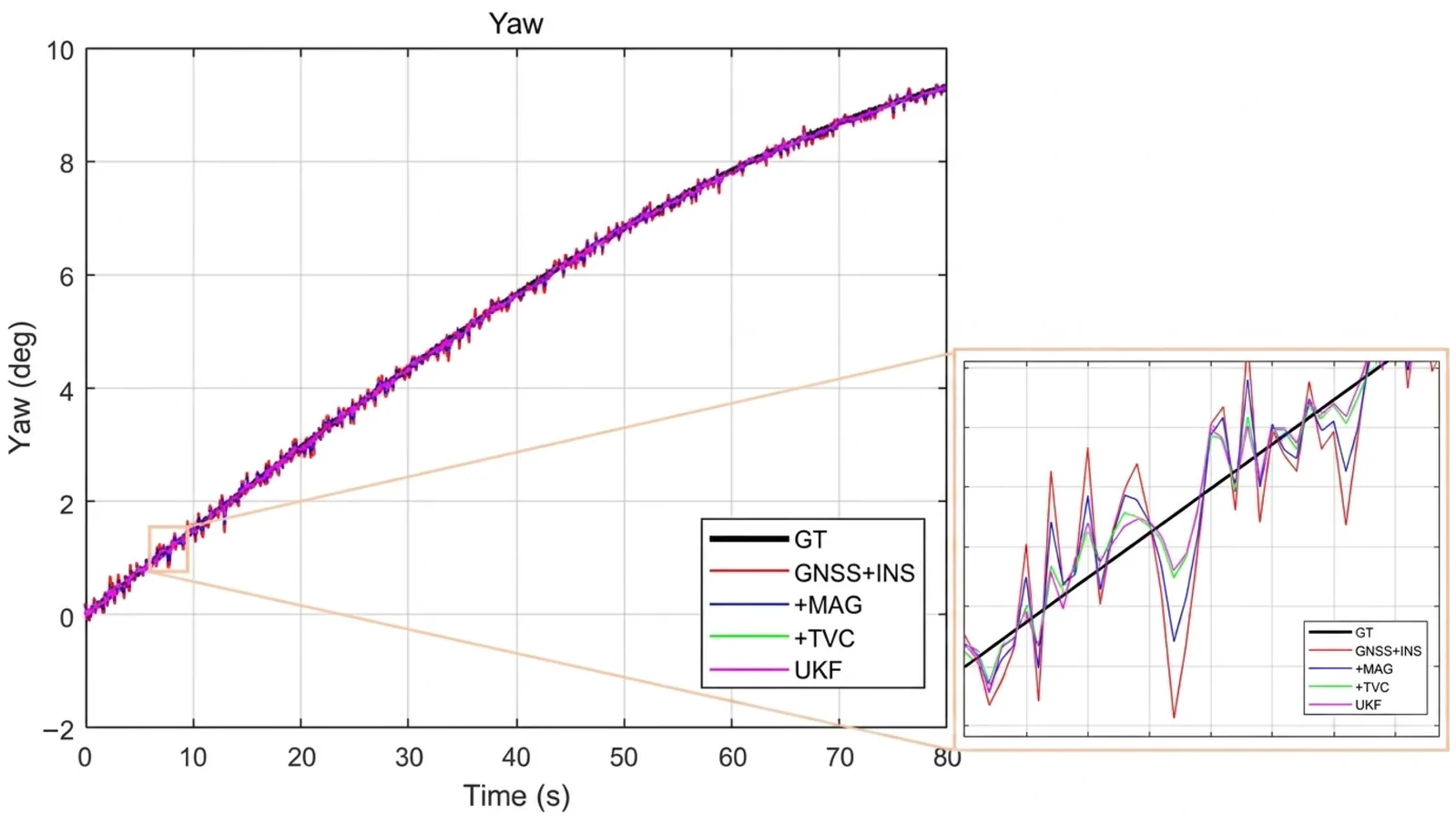
Yaw Angle Estimate.

**Figure 9:**
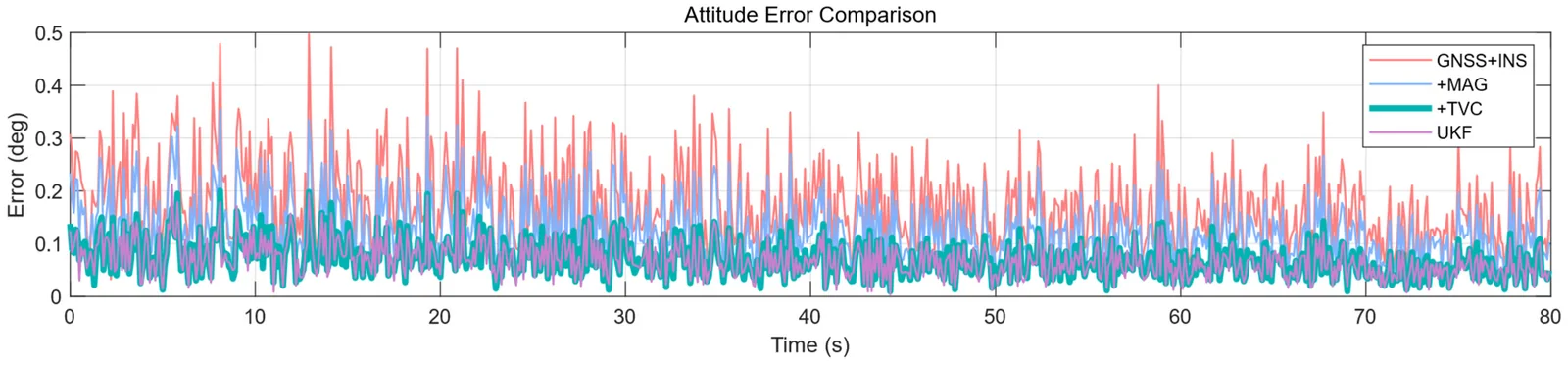
RMSE of all axis angles.

In addition, an Unscented Kalman Filter (UKF) is introduced as a nonlinear filtering benchmark. As shown in Figure 5, Figure 6, Figure 7, Figure 8 and Figure 9 and Table 2, UKF achieves slightly higher accuracy than the proposed IEKF in the nominal scenario (0.0789° vs. 0.0811°), but at a higher computational cost. The average per-step execution time is 0.32 ms for the IEKF versus 0.97 ms for the UKF (measured on an Intel Core i7-10700, 3.8 GHz, MATLAB R2023a), representing a 3× speed up for the proposed method. This computational advantage is particularly relevant for onboard implementation where timing constraints are stringent.

When the TVC model input is further introduced, the system performance is enhanced beyond what can be achieved through measurement updates alone. Unlike GNSS and magnetometer observations, which act as corrective measurements, the TVC input imposes constraints at the state propagation level, guiding system evolution through dynamic consistency. This structural constraint reduces prediction uncertainty and prevents the filter from relying solely on INS integration. As a result, the error growth rate is significantly reduced, and the estimated trajectory remains consistently closer to the ground truth throughout the entire simulation period, demonstrating superior accuracy.

### 3.3. Robustness Analysis

To ensure reproducibility of the robustness evaluation, the sensor anomaly scenarios are specified as follows. The dropout scenario simulates complete GNSS signal loss (position and velocity measurements unavailable) during a 10-s interval beginning at t=20 s, representing signal blockage or receiver tracking loss during ascent. The fault scenario simulates a slowly growing GNSS position bias: a ramp-type error increasing linearly from 0 to 50 m over 5 s (t=30–35 s) and then persisting at 50 m for the remainder of the simulation, representing satellite clock/ephemeris errors or ionospheric delays. For the IMU fault scenario, a gyroscope bias of $5^{\circ }/s$ is injected as a step change at t=40 s, simulating sensor saturation or internal failure. These scenarios are applied independently in separate Monte Carlo runs to isolate the effect of each anomaly type.

To evaluate robustness to TVC input uncertainty, we introduce Gaussian errors on the gimbal angles with standard deviations of $0.05^{\circ }$, $0.10^{\circ }$, and $0.20^{\circ }$, and thrust magnitude perturbations of 0.5%, 1.0%, and 2.0% of the nominal thrust. These values are representative of typical gimbal servo errors and chamber pressure fluctuations in liquid-propellant rocket engines. Under the highest uncertainty level ($0.20^{\circ }$ gimbal error and 2% thrust error), the RMSE increases from $0.0811^{\circ }$ to $0.0953^{\circ }$, demonstrating graceful degradation without filter divergence, as shown in Table 3.

The robustness analysis further evaluates system stability under sensor anomalies, considering both measurement dropout and faulty measurement scenarios. In the case of GNSS dropout, the GNSS+INS system rapidly degrades into a pure inertial navigation mode, where the state update relies entirely on INS outputs, leading to rapid error accumulation and significant degradation in stability. When the magnetometer is included, the system can still utilize heading information to partially constrain error growth during GNSS outages; however, in the presence of magnetometer faults, low-frequency biases may be absorbed by the filter, resulting in gradual drift or oscillatory behavior. This indicates that while the magnetometer improves robustness to some extent, it remains sensitive to persistent measurement errors.

**Figure 10:**
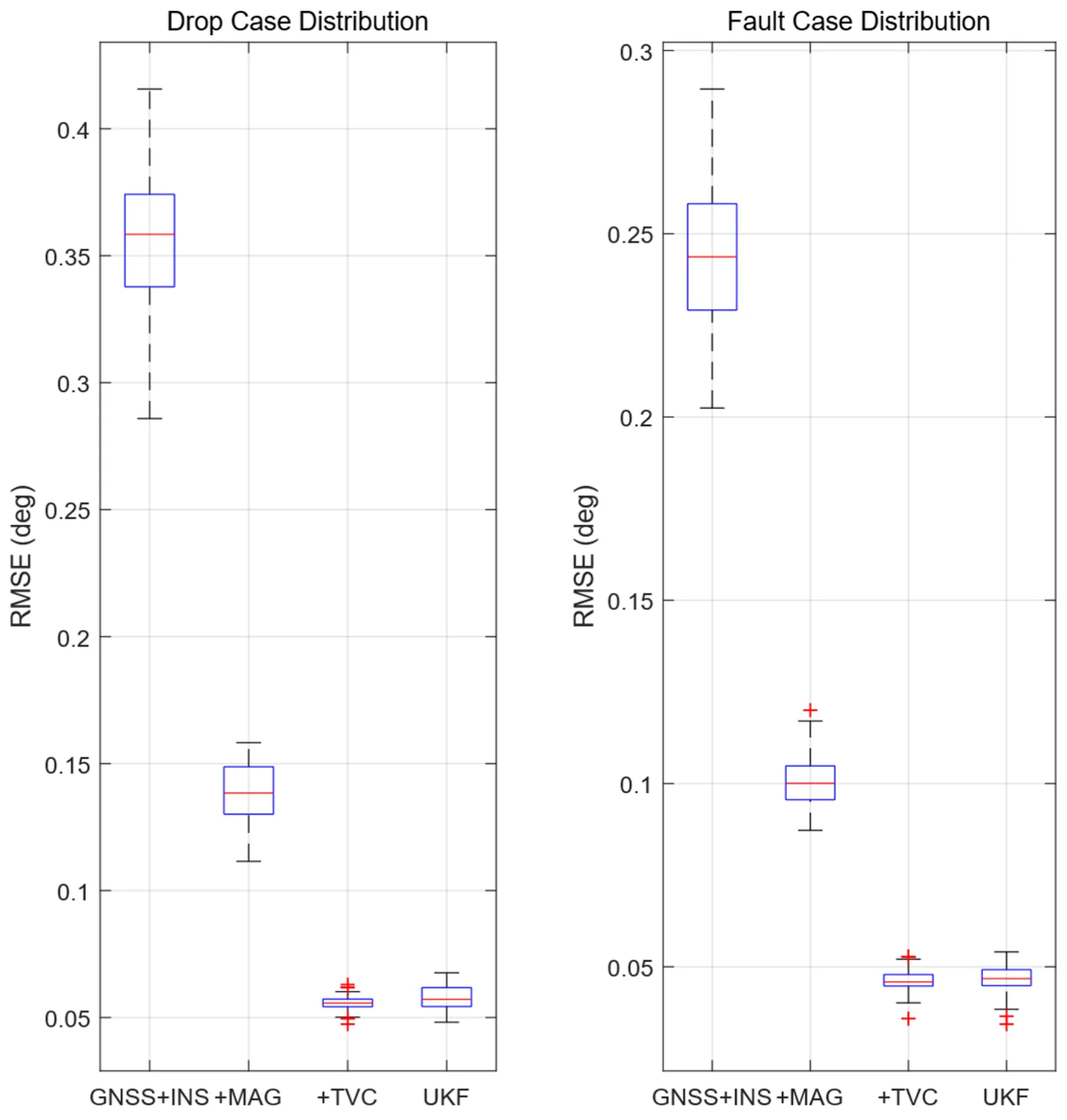
Drop and Fault RMSE distribution.

To further evaluate robustness under practical operating conditions, additional simulations are conducted by simultaneously introducing uncertainties in both the TVC actuation angles and IMU measurements. Specifically, the TVC input is corrupted by angular deflection errors, while the IMU outputs are affected by intermittent outliers and measurement perturbations. The corresponding Monte Carlo results are shown in Figure 10.

**Figure 11:**
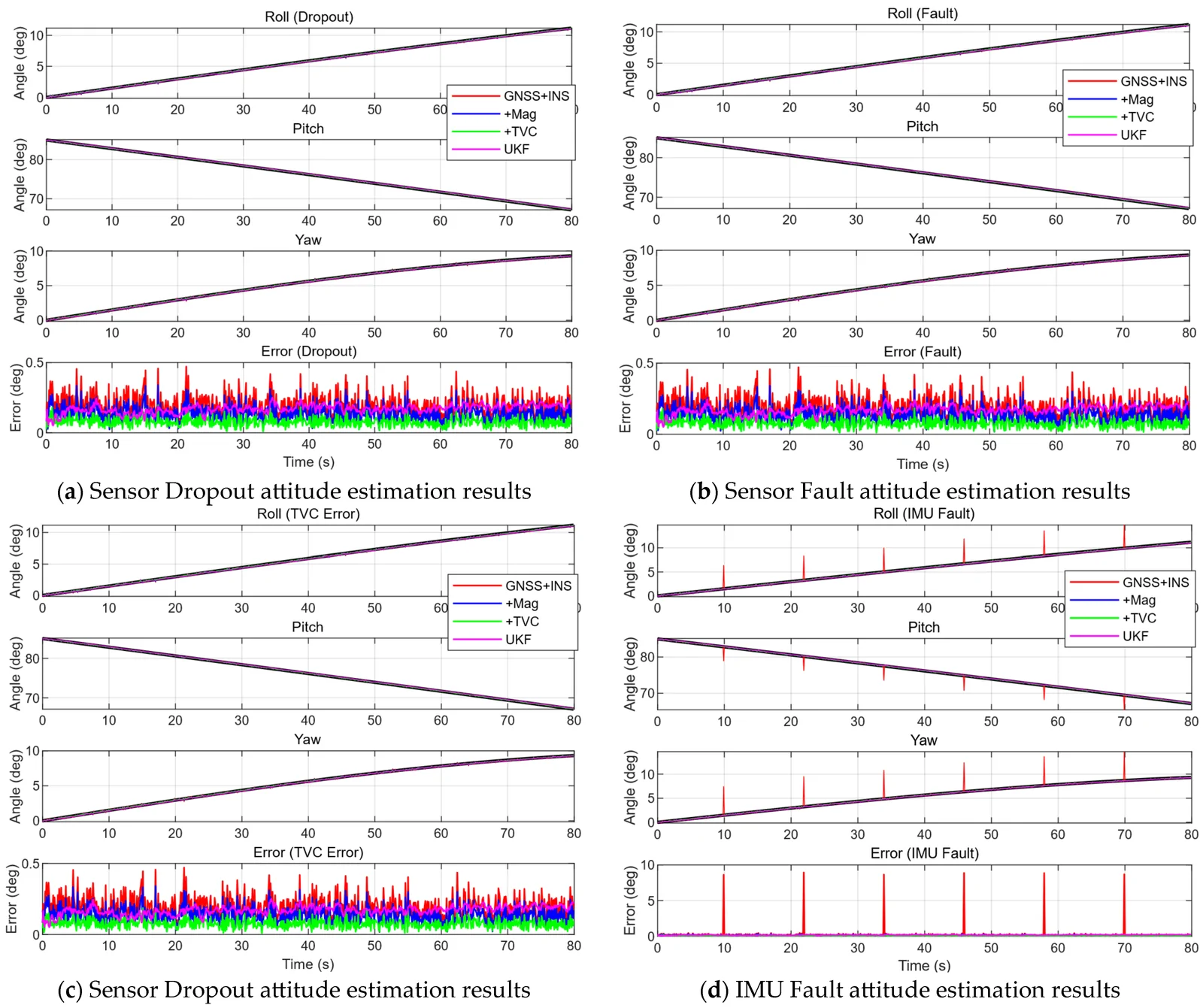
Robustness and attitude estimation under various fault conditions.

The results indicate that although estimation performance degrades under increased levels of combined uncertainties, the proposed framework maintains stable filtering behavior without divergence, as shown in Figure 11. This demonstrates that the TVC input acts as a structural constraint rather than a strictly deterministic control signal, while the filtering framework is also resilient to sporadic IMU measurement outliers. Consequently, the proposed method exhibits improved robustness in the presence of both actuator-level and sensor-level uncertainties.

In contrast, the inclusion of TVC inputs leads to a fundamentally different robustness characteristic. Even when GNSS or magnetometer measurements degrade or fail, the system can maintain stable state propagation by leveraging dynamic constraints. Although the TVC input does not provide direct external observations, it acts as a deterministic input that constrains the system evolution, thereby mitigating the unconstrained drift commonly observed in pure inertial systems. Consequently, the estimation error exhibits a suppressed growth trend rather than divergence. Furthermore, it is observed that measurement dropout generally has a more severe impact on system performance than faulty measurements, highlighting the critical role of continuous observation availability. In such scenarios, the stabilizing effect of TVC becomes particularly significant.

Nevertheless, it should be emphasized that when all external observations, including GNSS and magnetometer, are completely unavailable, a system relying solely on TVC inputs will still experience gradual error accumulation. However, the divergence rate remains significantly lower compared to a pure IMU-driven system. This observation suggests that TVC should not be considered a replacement for external sensors, but rather a structural constraint enhancement that complements existing observation sources. Its primary contribution lies in compensating for insufficient observations and improving both estimation accuracy and robustness under challenging operational conditions.

## 4. Discussion

The results demonstrate that incorporating TVC as a deterministic input significantly improves attitude estimation accuracy and robustness compared with conventional GNSS/INS and GNSS/INS/MAG fusion. The hierarchical RMSE reduction, from 0.1966° to 0.1443° with the addition of magnetometer, and further to 0.0811° with TVC integration, reveals distinct yet complementary mechanisms: the magnetometer primarily constrains yaw drift by providing absolute heading observations, while TVC imposes dynamic consistency at the propagation level, effectively reducing prediction uncertainty and bounding INS integration error growth. This distinction is particularly evident in the dropout scenarios (Table 2), where the TVC-aided configuration maintains an RMSE of 0.0555° compared with 0.1384° for the magnetometer-aided case, indicating that dynamic constraints offer a fundamentally different form of robustness than additional measurements alone.

The improved performance can be attributed to two factors. First, the right-invariant error formulation on SE_2_(3) ensures that the error dynamics for the dominant rotational and translational states are independent of the actual trajectory, avoiding the inconsistency issues that plague conventional EKF when linearizing around large attitude errors. Second, by explicitly feeding TVC commands into the state predictor, the filter exploits the physical coupling between the thrust direction and acceleration of launch vehicle, which enhances the observability of the attitude states, particularly yaw, as formally shown in the observability analysis in Section 2.7.

Compared with the UKF benchmark, the proposed IEKF achieves comparable accuracy (0.0811° vs. 0.0789° in the normal scenario) with significantly lower computational cost. The UKF requires the propagation of 43 sigma points through the nonlinear dynamics, whereas the IEKF propagates only a single nominal state with a linearized covariance update. This computational advantage is especially relevant for launch vehicle onboard implementation, where processing power and timing constraints are stringent.

Several limitations of the current study should be acknowledged. The flat-Earth assumption restricts the formulation to the early ascent phase; the random-walk disturbance model may not fully capture rapidly varying aerodynamic forces; and the TVC inputs are treated as deterministic commands, whereas actuator dynamics introduce uncertainty in practice. The sensitivity analysis in Section 3.3 provides a preliminary assessment of these effects, but explicit noise propagation for control inputs would further strengthen robustness. These limitations, together with the need for real flight data validation, constitute the primary directions for our future work.

## 5. Conclusions

This paper has presented a TVC-aided invariant extended Kalman filter for launch vehicle attitude estimation. By explicitly incorporating thrust vector control commands into the IEKF propagation step and formulating the error on the SE_2_(3) Lie group, the proposed method achieves consistent and robust attitude estimation while exploiting the physical coupling between control inputs and launch vehicle dynamics. The main contributions of this work are threefold: (1) a novel TVC-aided IEKF formulation that models the coupling between TVC and launch vehicle attitude within a Lie-group-consistent framework; (2) a rigorous observability analysis demonstrating improved system observability under TVC-active conditions; (3) comprehensive simulation validation showing significant improvements over conventional EKF and UKF benchmarks. Despite these promising results, several limitations should be acknowledged—including the flat-Earth assumption, the simplified disturbance model, the idealization of TVC inputs, and the lack of real flight data validation—which suggest meaningful future directions, particularly extending the framework to full-trajectory applications, incorporating adaptive disturbance models, and validating the method with real flight data from an upcoming sounding rocket campaign.

## Figures and Tables

**Table 1:** Key Simulation Parameters and Filter Hyperparameters.

Parameter	Value
IMU (Gyroscope)	
Measurement range	$\pm 300^{\circ }/s$
Bias instability	$0.03^{\circ }/h$ (1σ)
Angle random walk	$0.005^{\circ }/\sqrt{h}$
Sampling rate	100 Hz
IMU (Accelerometer)	
Measurement range	±30 g
Bias instability	3 μg (1σ)
Velocity random walk	$0.01\;m/s/\sqrt{h}$
GNSS	
Position noise	10 m (3σ)
Velocity noise	0.1 m/s (3σ)
Update rate	10 Hz
Magnetometer	
Noise	100 nT
Update rate	20 Hz
Initial Uncertainties	
Attitude	$15^{\circ }$ (3σ)
Position	1 m (3σ)
Velocity	0.1 m/s (3σ)
Gyroscope bias	$0.1^{\circ }/h$ (3σ)
Accelerometer bias	50 μg (3σ)
Filter Parameters	
IEKF iterations	5
UKF α	$10^{-3}$
UKF β	2

**Table 2:** Attitude Estimation Accuracy Analysis.

Algorithm	RMSE (°)
GNSS+INS	0.1966
GNSS+INS+MAG	0.1443
GNSS+INS+MAG+TVC	0.0811
GNSS+INS+MAG+TVC(UKF)	0.0789

**Table 3:** Attitude Estimation Robustness Analysis.

Algorithm	RMSE (°)	
	Drop	Fault
GNSS+INS	0.3526	0.2432
GNSS+INS+MAG	0.1384	0.1003
GNSS+INS+MAG+TVC	0.0555	0.0454
GNSS+INS+MAG+TVC (UKF)	0.0582	0.0467

## Data Availability

The data supporting this study cannot be publicly shared due to privacy. Further inquiries about the data should be directed to the corresponding author.
